# Metastatic breast cancer: prolongation of survival in routine care is restricted to hormone-receptor- and Her2-positive tumors

**DOI:** 10.1186/2193-1801-3-535

**Published:** 2014-09-17

**Authors:** Rudolf Weide, Stefan Feiten, Vera Friesenhahn, Jochen Heymanns, Kristina Kleboth, Jörg Thomalla, Christoph van Roye, Hubert Köppler

**Affiliations:** Praxisklinik für Hämatologie und Onkologie, Neverstr. 5, 56068 Koblenz, Germany; Institut für Versorgungsforschung in der Onkologie, Neverstr. 5, 56068 Koblenz, Germany

**Keywords:** Breast cancer, Metastasis, Outpatient treatment, Overall survival, Retrospective study

## Abstract

18,000 women die due to metastatic breast cancer in Germany per year. Median survival is 20–28 months after diagnosis. The question we wanted to answer was whether survival has improved in routine care? For this purpose we conducted a retrospective analysis of all patients with metastatic breast cancer who were treated between 06/1995-06/2013 in a community-based oncology group practice in Germany. 716 patients were analyzed with a median age of 61 (31–93). Localizations of metastases were distributed as follows: 47% visceral, 36% bone, 9% lymphatic, 4% CNS, 4% others. 79% were hormone-receptor-positive, 20% Her2-positive, 9% triple-negative. Median overall survival was 34 months (95% Confidence Interval: 31–37), median disease-specific survival 36.8 months and disease-specific survival after 5 years 34%. Survival was significantly correlated with localizations of metastases, number of metastasized organs, disease free survival since initial diagnosis, hormone-receptor status and age. Patients with hormone-receptor-positive tumors had a median overall survival of 37 months, Her2-positive patients of 34 months and triple-negative patients of 13 months. 86% of hormone-receptor-positive patients received antihormonal therapy. 81% of Her2-positive patients received anti-Her2 therapy. In summary, longer survival is strongly restricted to hormone receptor- and Her2-positive tumors most likely due to targeted therapies directed against the estrogen-receptor and Her2.

## Introduction

Metastatic breast cancer is the tumor with the highest mortality in women. In Germany about 18,000 women die from breast cancer annually (Statistisches Bundesamthttps://www.destatis.de/DE/ZahlenFakten/GesellschaftStaat/Gesundheit/Todesursachen/Tabellen/SterbefaelleInsgesamt.html). The median survival after initial diagnosis is 20–28 months (Mauri et al. 2008; Gennari et al. 2005; Giordano et al. 2004; Chia et al. 2007; Tai et al. 2004; Dafni et al. 2010; Tevaarwerk et al. 2013; Allemani et al. 2013). The probability to be alive 5 years after initial diagnosis of metastasis is according to tumor registry data from Germany, England and the USA only 13-27% (Tumorregister Münchenhttp://www.tumorregister-muenchen.de/facts/surv/surv_C50f_G.pdf; Holleczek et al. 2011; Weide et al. 2012; Office for National Statistics (ONS)http://www.ons.gov.uk/ons/publications/re-reference-tables.html?edition=tcm%3A77-241176; Surveillance, Epidemiology and End Results Program (SEER 18, 2004–2010)http://seer.cancer.gov/statfacts/html/breast.html#survival). During the last 18 years advances in targeted therapy have been described in hormone-receptor and Her2-positive tumors, which have resulted in prospective trials in longer survival. With regard to hormone-receptor-positive tumors the introduction of aromatase inhibitors, the antiestrogen fulvestrant and the mTOR inhibitor everolimus has to be mentioned (Montemurro et al. 2012). The discovery of Her2 as a negative prognostic factor and the associated development of trastuzumab and other anti-Her2 therapies have led in studies to a significant improvement in survival of these patients (Murphy & Morris 2012). Tumor registry data from the Saarland and from Munich imply that the survival of patients with metastatic breast cancer has not improved in the last 20–30 years in Germany (Holleczek et al. 2011; Schlesinger-Raab et al. 2005). Contradictory publications are available from other tumor registries and other institutions that describe a survival improvement in routine care (Mauri et al. 2008; Gennari et al. 2005; Giordano et al. 2004; Chia et al. 2007; Tai et al. 2004; Dafni et al. 2010; Allemani et al. 2013; Weide et al. 2009). This improvement in survival is causally attributed by the authors to the introduction of new drugs (cytostatic agents, antihormonal therapy and anti-Her2 therapies) (Mauri et al. 2008; Gennari et al. 2005; Giordano et al. 2004; Chia et al. 2007; Dafni et al. 2010; Allemani et al. 2013; Weide et al. 2009).

The objective of the present study was to find out whether survival of unselected patients with metastatic breast cancer has improved in routine care and if survival differences can be detected?

## Patients and methods

Retrospective analysis of all patients with metastatic breast cancer who were treated between 06/1995-06/2013 in an oncology group practice (Praxisklinik für Hämatologie und Onkologie Koblenz, Germany). All relevant data were transferred from patient files into a database and statistically analyzed using SPSS 19 and SURVSOFT.

At initial diagnosis the following data, among others, were collected: TNM staging, estrogen- and progesterone-receptor status, Her2 status (positively defined as IHC 3+ or IHC 2+ and CISH/FISH-positive) and adjuvant therapy.

At the time of metastasis was captured: time from initial diagnosis to metastasis, age, estrogen- and progesterone-receptor status, Her2 status, menopausal status, comorbidities (Charlson Score), metastatic sites, number of involved organs, palliative therapies, hospitalizations, causes and places of death and overall survival (time from initial diagnosis of metastasis to death or end of observation).

Multivariate and univariate survival analyses were conducted. First of all patients were classified according to metastatic sites at initial diagnosis as follows: CNS affection, visceral metastasis without CNS affection, regional metastasis of the lymph nodes, bone only metastasis and localizations which could not be classified in this scheme. For each group a median survival was calculated with the help of the Kaplan-Meier method and differences in survival were tested for statistical significance by log-rank-tests. Analyses concerning receptor status were carried out analogically. Patients were either Her2-positive or/and hormone-receptor positive or triple-negative.

The following potential parameters were dichotomized tested in univariate Kaplan-Meier analyses and log-rank tests: bone metastasis, metastasis of the lymph nodes, visceral metastasis, CNS affection, hormone-receptor status, Her2-status, menopausal status and primary metastasis. An univariate Cox regression was performed to investigate the influence of the following quantitative or semi-quantitative variables: Charlson Score, age at initial diagnosis of metastasis, time between diagnosis of breast cancer and metastasis in months and number of metastatic sites. An adjustment for multiple testing was applied.

All parameters that were found to be influential in the univariate analyzes were tested in a multifactorial Cox regression. The following parameters were included in a forward stepwise modeling process: hormone-receptor status, CNS affection, visceral localizations, age at initial diagnosis of metastasis, time between diagnosis of breast cancer and metastasis in months and number of metastatic sites.

To evaluate a development of survival over time, roughly equal quartiles of the diagnosis periods were generated: before 2000, 01/2000-12/2003, 01/2004-12/2007, 01/2008-06/2013. For these quartiles, median overall and disease-specific survival was calculated and tested for statistical significance.

All patients gave written informed consent concerning analysis and publication of data.

## Results

### Patient characteristics

716 female patients were treated between 06/1995-06/2013 (see Table 1). Median age at diagnosis of metastasis was 61 years (31–93), 80% (n = 572) were postmenopausal. Patients suffered from few comorbidities, mean Charlson Score was 0.3 (0–5). Localizations of metastases were distributed as follows: 47% (n = 335) visceral, 36% (n = 256) bone, 9% (n = 66) lymph nodes, 4% (n = 29) CNS and 4% (n = 29) others. The mean number of involved organs was 1.4 (1–4). 79% of patients (n = 563) were hormone-receptor-positive, 20% (n = 145) Her2-positive and 9% (n = 62) triple-negative. In 7% of patients (n = 47) the receptor status could not be determined retrospectively.

**Table 1:** **Patient characteristics**

Adjuvant therapy [multiple responses possible]		
- chemotherapy	42%	n = 303
- antihormonal therapy	45%	n = 320
- radiation	47%	n = 333
Disease free survival from initial diagnosis of breast cancer to metastasis		
- median	32.5 months	
- < 24 months	42%	n = 303
- ≥ 24 months	58%	n = 413
Age at diagnosis of metastasis		
- median	61 years	
- < 50 years	20%	n = 146
- 50–70 years	56%	n = 402
- >70 years	23%	n = 168
Menopausal status at time of metastasis		
- premenopausal	19%	n = 134
- postmenopausal	80%	n = 572
- not evaluable	1%	n = 10
Hormone-receptor status		
- negative	16%	n = 117
- positive	79%	n = 563
- not evaluable	5%	n = 36
Her2 status		
- negative	52%	n = 369
- positive	20%	n = 145
- not evaluable	28%	n = 202
Comorbidities at time of metastasis		
- Charlson Score 0	81%	n = 578
- Charlson Score 1 + 2	17%	n = 123
- Charlson Score >2	2%	n = 13
Metastatic status at initial diagnosis of breast cancer		
- M1	21%	n = 151
- M0	76%	n = 547
- not evaluable	3%	n = 18
Localizations of metastases at initial diagnosis of metastasis		
- visceral	47%	n = 335
- bone	36%	n = 256
- CNS	4%	n = 29
- lymph nodes	9%	n = 66
- others	4%	n = 29
Number of involved organs at initial diagnosis of metastasis		
- 1 localization	69%	n = 493
- >1 localizations	31%	n = 223

### Palliative therapy

98% of patients (n = 701) received palliative therapy, 2% (n = 15) solely best supportive care. A total of 3,346 palliative therapies were carried out, the median number of different lines per patient was 4 (0–20). First line therapy consisted of endocrine treatment in 58% (n = 414). Agents used were aromatase inhibitors in 74% (n = 308) and tamoxifen in 21% (n = 88). First line therapy was chemotherapy in 40% (n = 284). Agents most frequently used were anthracyclines in 29% (n = 82), taxanes in 24% (n = 68), capecitabine in 14% (n = 40) and anthracycline/taxane-combinations in 11% (n = 31).

### Antihormonal therapies

86% (n = 484) of patients with hormone-receptor-positive tumors (n = 563) received an antihormonal therapy in the course of their disease. 89% (n = 429) of them received an aromatase inhibitor, 37% (n = 181) fulvestrant, 28% (n = 135) tamoxifen, 12% (n = 56) a LHRH agonist and 3% (n = 14) everolimus.

### Anti-Her2 therapies

81% (n = 118) of all Her2-positive patients (n = 145) received an anti-Her2 therapy in the course of the disease. 98% (n = 116) of them were treated with trastuzumab, 23% (n = 27) with lapatinib, 6% (n = 7) with TDM-1 and 2% (n = 2) with trastuzumab + pertuzumab.

### Chemotherapy and bevacizumab

83% of patients (n = 594) received at least one chemotherapy line (0–17). Most frequently applied were anthracyclines (mitoxantrone or epirubicin or doxorubicin) in 64% (n = 381), taxanes (58%, n = 346), capecitabine (54%, n = 323) and vinorelbine (43%, n = 257). 16% (n = 93) were treated with bevacizumab.

### Bone protecting therapy

87% (n = 412) of patients with bone metastases (n = 475) were treated with a bone protecting therapy. 94% (n = 388) of them received a bisphosphonate, 5% (n = 22) the monoclonal RANK ligand inhibitor denosumab. The rate of treatment-associated osteonecrosis of the jaw was 4% (n = 16).

### Treatment of brain metastases

At the time of diagnosis of metastasis 3% (n = 25) already had one or more brain metastases. In the course of the disease 18% (n = 132) of the patients developed brain metastases. The treatment of brain metastases consisted of whole brain radiation in 73% (n = 96), surgical resection of metastases in 16% (n = 21) and intrathecal MTX therapy in 5% (n = 7). In 17% of patients (n = 23) no therapy of brain metastases was performed.

### Toxicities and hospitalizations

Overall, grade 3 and grade 4 toxicities were low at 12% of all 3,346 therapies. No treatment-associated death was observed. After diagnosis of metastasis 82% (n = 584) of patients had to be hospitalized during treatment. For all patients the median frequency of hospitalizations was 2 (0–18), the median duration per hospital stay was 8 days (1–79). The median cumulative duration of hospitalization in an acute care hospital was 18 days (1–173). The median duration of the last hospitalization before death was 6 days (1–57). 71% (n = 508) of patients had to be hospitalized due to breast cancer, 31% (n = 223) because of comorbidities and only 6% (n = 40) due to side effects of palliative therapy.

### Causes and places of death

At the end of the evaluation period (06/2013) 77% (n = 549) of patients had died. 37% (n = 202) were able to die at home, 42% (n = 228) died in hospital, 6% (n = 31) in nursing homes, 5% (n = 26) in hospice care. For 11% (n = 62) the place of death could not be determined. 82% (n = 451) of the patients died of their tumor, 8% (n = 46) of comorbidities. For 9% (n = 52) the cause of death could not be determined. Treatment-related deaths were not observed.

### Survival

**Figure 1:**
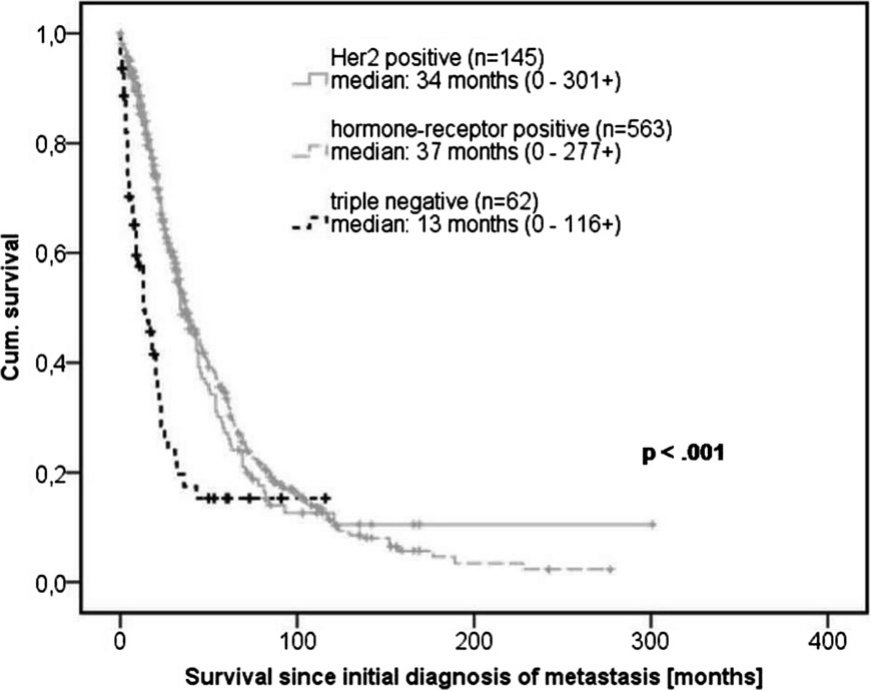
**Overall survival depending on the receptor status.**

Median survival of all patients from initial diagnosis of metastasis was 34 months (95% Confidence Interval [CI]: 31–37), disease-specific survival was 36.8 months. The disease-specific survival after 5 years was 34%, 12% after 10 years and 6% after 15 years.Significant differences (p < 0.01) in overall survival were observed depending on localizations of metastases at initial diagnosis. Patients with lymph node metastases lived 47 months (95% CI: 36–58) in median, patients with solely bone metastases 43 months (95% CI: 36–50), patients with visceral metastases 26 months (95% CI: 23–29) and patients with CNS metastases only 11 months (95% CI: 0–23). A significant survival difference could also be found depending on the receptor status (p < 0.01). Patients with hormone-receptor-positive tumors survived 37 months (95% CI: 33–41) in median, Her2-positive patients 34 months (95% CI: 27–41), triple-negative patients only 13 months (95% CI: 6–20) (see Figure 1). No significant survival difference was observed between patients with primary metastases and patients whose metastases had occurred after adjuvant therapy.

Median disease-specific survival as a function of diagnosis period was as follows: 32.7 months (before 2000), 36.9 months (01/2000-12/2003), 39.1 months (01/2004-12/2007) and 34.5 months (01/2008-06/2013). Differences in survival did not reach statistical significance. More than 90% of patients who were diagnosed before 12/2003 had died, 78% in the diagnosis period 01/2004-12/2007 and 46% in the last cohort.

As significant in terms of survival proved in the multivariate approach the following parameters: CNS metastases, positive hormone-receptor status, age at initial diagnosis of metastasis, time between initial diagnosis of breast cancer and metastasis in months and number of metastatic localizations. The presence of CNS metastases had the strongest impact on survival, whereas the presence of visceral metastases failed to reach statistical significance. For patients with CNS metastases the risk of dying was higher by 148% in comparison to patients without CNS metastases. Of high predictive value was also the hormone-receptor status. Patients with a positive hormone-receptor status had a 40% lower risk of dying than patients with a negative status. For the number of metastatic localizations could be established that the risk of death for each additional localization increased by 18%. The age at the time of metastasis and the time between initial diagnosis of breast cancer and metastasis had a small, albeit statistically significant influence on survival. The longer the time between initial diagnosis of breast cancer and metastasis was, the longer the survival from metastasis, namely 0.4% per month (see Table 2).

**Table 2:** **Results of the Cox regression**

	Significance	Hazard ratio	95% confidence interval for hazard ratio	
			Lower limit	Upper limit
Hormone-receptor status positive	p < 0.001	0.599	0.475	0.755
CNS metastases	p < 0.001	2.479	1.547	3.972
Age at initial diagnosis of metastasis [years]	p < 0.001	1.015	1.007	1.022
Time [months] between initial diagnosis and metastasis	p < 0.001	0.996	0.995	0.998
Number of metastatic localizations	p = 0.010	1.182	1.041	1.342

## Discussion

Treatment goals of women with metastatic breast cancer are prolongation of life and preservation of quality of life.

### Prolongation of life

Median survival of patients with metastatic breast cancer is 20–28 months (Mauri et al. 2008; Gennari et al. 2005; Giordano et al. 2004; Chia et al. 2007; Tai et al. 2004; Dafni et al. 2010; Tevaarwerk et al. 2013; Allemani et al. 2013) from diagnosis of metastasis. Disease-specific survival after 5 years in breast cancer registries is 13% in England, 20-27% in Germany and 23-24% in the USA (Tumorregister Münchenhttp://www.tumorregister-muenchen.de/facts/surv/surv_C50f_G.pdf; Holleczek et al. 2011; Weide et al. 2012; Office for National Statistics (ONS)http://www.ons.gov.uk/ons/publications/re-reference-tables.html?edition=tcm%3A77-241176; Surveillance, Epidemiology and End Results Program (SEER 18, 2004–2010)http://seer.cancer.gov/statfacts/html/breast.html#survival). The survival of patients in prospective clinical trials is very different due to necessary selection criteria and different research objectives. We also know that only about 2% of patients who are older than 60 are treated within clinical trials (American College of Physicians (ACP)http://www.acponline.org/products_services/mksap/16/). Therefore, comparisons between study results and routine care are very limited. Recent reports from the Munich cancer registry (Schlesinger-Raab et al. 2005) and the tumor registry of the Saarland (Holleczek et al. 2011) show no improvement in survival in patients with metastatic breast cancer in the last 20–30 years. In contrast, there are a variety of publications from individual institutions and tumor registries that describe a prolongation of survival time in the last 20–30 years (Mauri et al. 2008; Gennari et al. 2005; Giordano et al. 2004; Chia et al. 2007; Tai et al. 2004; Dafni et al. 2010; Allemani et al. 2013; Weide et al. 2009). Our analysis of more than 700 unselected, consecutive patients in routine care confirms a prolongation of survival with a median overall survival of 34 months, a disease-specific survival of 36.8 months and a disease-specific survival of 34% after 5 years. This comparatively long survival in our population, however, is limited to patients with hormone-receptor- or Her2-positive tumors most likely due to targeted therapies directed against the estrogen-receptor and Her2.

### Hormone-receptor-positive tumors

The current guidelines recommend for hormone-receptor-positive tumors an antihormonal first-line therapy, if so permitted by the clinical situation (Arbeitsgemeinschaft Gynäkologische Onkologie (AGO)http://www.ago-online.de/de/fuer-mediziner/leitlinien/mamma/; Onkopediahttps://www.dgho-onkopedia.de/de/mein-onkopedia/leitlinien/brustkrebs-der-frau). In prospective clinical trials it could be demonstrated that aromatase inhibitors are more effective than tamoxifen in the antihormonal first-line treatment of metastatic breast cancer in postmenopausal patients (Nabholtz et al. 2000; Mouridsen et al. 2003). The introduction of the new antiestrogen fulvestrant demonstrated superiority to the aromatase inhibitor anastrozole (Robertson et al. 2012). In a recent study, the superiority of a combination of fulvestrant plus anastrozole compared to a sole anastrozole therapy could be shown (Mehta et al. 2012).

After resistance to a nonsteroidal aromatase inhibitor, the combination of the steroidal aromatase inhibitor exemestane plus the mTOR inhibitor everolimus showed a significantly prolonged progression-free survival compared with exemestane alone (Baselga et al. 2012). A meaningful sequence in postmenopausal patients is the first-line therapy with a nonsteroidal aromatase inhibitor, followed by second-line treatment with fulvestrant, followed by third-line therapy with the steroidal aromatase inhibitor exemestane plus everolimus. If it is permitted by the clinical situation patients should only then, in fourth line, be treated with a chemotherapy.

Premenopausal patients should always be treated in first line with an inhibition of ovarian function (LHRH agonist) and tamoxifen or with an oophorectomy and an aromatase inhibitor (Arbeitsgemeinschaft Gynäkologische Onkologie (AGO) http://www.ago-online.de/de/fuer-mediziner/leitlinien/mamma/; Onkopediahttps://www.dgho-onkopedia.de/de/mein-onkopedia/leitlinien/brustkrebs-der-frau). In our population 79% of patients (n = 563) had a tumor that was estrogen- and/or progesterone-receptor positive. 86% of these patients (n = 484) received an antihormonal therapy. Of these, 89% (n = 429) were treated with an aromatase inhibitor, 37% (n = 181) with fulvestrant, 28% (n = 135) with tamoxifen, 12% (n = 56) with a LHRH agonist and 3% (n = 14) with exemestane plus everolimus. In our cohort the combination of exemestane plus everolimus can't be made responsible for a prolongation of survival because this combination was licensed in Germany just a couple of months ago. Hormone-receptor-positive patients had a median overall survival of 37 months. Thus our results imply most likely that the sequential use of the most potent antihormonal therapies is the basis for long-term survival in hormone-receptor-positive tumors in routine care.

### Her2-positive tumors

Her2-positive tumors are characterized by increased proliferation, invasiveness and metastasis tendency (Slamon et al. 1987). The median survival in Her2-positive tumors is without anti-Her2 therapy only 18 months (Slamon et al. 1987; Marty et al. 2005). The monoclonal anti-Her2 antibody trastuzumab led with paclitaxel or anthracycline chemotherapy to a prolongation of survival from 20.3 to 25.1 months (Slamon et al. 2001). Even in progressive course under paclitaxel plus trastuzumab, it is useful to maintain trastuzumab and convert only the chemotherapy partner to a non-cross-resistant substance (von Minckwitz et al. 2009). An alternative in tumor progression on trastuzumab offers the tyrosine kinase inhibitor lapatinib, which is approved in Germany in combination with capecitabine for this indication. Lapatinib has the advantage that it is effective even in brain metastases due to its ability to pass into the liquor (Lin et al. 2008). Recently the dual Her2 blockade with lapatinib and trastuzumab for patients suffering from metastatic Her2-positive, hormone-receptor-negative breast cancer was approved. A further development is the new anti-Her2 antibody pertuzumab which prevents the heterodimerization between Her2 and Her3. Thus the intracellular tyrosine kinase is not activated, which is responsible for the initiation of the activation cascade.

In the CLEOPATRA-trial it could be proved that the dual inhibition of Her2 by trastuzumab and pertuzumab in combination with docetaxel chemotherapy prolonged survival significantly compared with the combination of docetaxel plus trastuzumab (Swain et al. 2013). Another new development is the anti-Her2 antibody TDM-1, where emtansine, a highly effective microtubule inhibitor, is coupled with trastuzumab. Through the internalization of Her2 plus TDM-1 emtansine enters the Her2-positive cell and can induce a selective cell death. In the EMILIA trial it could be demonstrated that TDM-1 is more effective than lapatinib plus capecitabine in tumor progression on a trastuzumab-containing regimen (Verma et al. 2012). In our collective 20% (n = 145) of patients suffered from Her2-positive tumors. In the course of treatment 81% (n = 118) of these patients received an anti-Her2 therapy (98% (n = 116) trastuzumab, 23% (n = 27) lapatinib, 6% (n = 7) TDM-1 and 2% (n = 2) trastuzumab + pertuzumab)). In our analysis pertuzumab and TDM-1 can't be made responsible for a prolongation of survival because they were licensed in Germany as recently as 2013. The overall survival of Her2-positive patients was 34 months and thus emphasizes the importance of a targeted anti-Her2 therapy in routine care of patients with metastatic breast cancer.

### Triple-negative tumors

9% of patients (n = 62) suffered from a tumor which was neither hormone-receptor- nor Her2-positive. Triple-negative tumors are characterized by a very aggressive course and a high metastatic tendency. In studies, the median survival is only 9–11 months (Carey et al. 2012). In our collective, the median survival was only 13 months, well below that of the hormone-receptor- or Her2-positive tumors. Neoadjuvant studies presented at the San Antonio Breast Cancer Symposium 2013 suggest that treatments using carboplatin and PARP-inhibitors like veliparib may be especially effective in triple negative tumors (Sikov et al. 2013; Rugo et al. 2013). These treatments may be used successfully in the metastatic situation as well.

### Preservation of quality of life

The preservation of quality of life is the second major goal in the treatment of patients with metastatic breast cancer. The concept of an oncology group practice makes it possible to treat patients close to residence at a high professional level. It seems important to us to point out in this connection the continuity and constancy of care of patients and their relatives by the oncology team. In an own analysis we found a mean psychosocial distress level of only 5.2 for patients with metastatic breast cancer who were treated in our practice (Mergenthaler et al. 2011). This value was determined in a cross-sectional survey using the NCCN Distress Thermometer. Another important factor for holistic care is in our opinion the double qualification as medical oncologist and palliative care physician since throughout the course of the disease, with varying intensity, always palliative care aspects come to the fore.

The strengths of this evaluation are the high number of patients treated over 18 years, the completeness of treatment data and the fact that no patients were excluded from the analysis. Potential drawbacks of the study are that this is a retrospective unicenter analysis and that we have no standardized data concerning best response and progression-free-survival.

## Conclusion

In conclusion, the treatment of unselected patients with metastatic breast cancer in an oncology group practice leads to a prolonged overall survival compared with registry data from Germany, England and the United States. Prolongation of survival was restricted to patients with hormone-receptor- and Her2-positive tumors most likely due to targeted therapies directed against the estrogen receptor and Her2.

### Ethical standards

According to the ethical review committee of Rhineland-Palatinate an approval was not necessary due to the retrospective analysis of routine care data.

## References

[CR1] Allemani C, Minicozzi P, Berrino F, Bastiaannet E, Gavin A, Galceran J, Ameijide A, Siesling S, Mangone L, Ardanaz E, Hédelin G, Mateos A, Micheli A, Sant M, EUROCARE Working Group (2013). Predictions of survival up to 10 years after diagnosis for European women with breast cancer in 2000–2002. Int J Cancer.

[CR2] American College of Physicians (ACP): **Medical Knowledge Self Assessment Program® (MKSAP®): Hematology and Oncology.** http://www.acponline.org/products_services/mksap/16/. Accessed 20 May 2013

[CR3] Arbeitsgemeinschaft Gynäkologische Onkologie (AGO): **Leitlinien/Empfehlungen: Mamma Version 13.1.0.** http://www.ago-online.de/de/fuer-mediziner/leitlinien/mamma/. Accessed 17 May 2013.

[CR4] Baselga J, Campone M, Piccart M, Burris HA, Rugo HS, Sahmoud T, Noguchi S, Gnant M, Pritchard KI, Lebrun F, Beck JT, Ito Y, Yardley D, Deleu I, Perez A, Bachelot T, Vittori L, Xu Z, Mukhopadhyay P, Lebwohl D, Hortobagyi GN (2012). Everolimus in postmenopausal hormone-receptor-positive advanced breast cancer. N Engl J Med.

[CR5] Carey LA, Rugo HS, Marcom PK, Mayer EL, Esteva FJ, Ma CX, Liu MC, Storniolo AM, Rimawi MF, Forero-Torres A, Wolff AC, Hobday TJ, Ivanova A, Chiu WK, Ferraro M, Burrows E, Bernard PS, Hoadley KA, Perou CM, Winer EP (2012). TBCRC 001: randomized phase II study of cetuximab in combination with carboplatin in stage IV triple-negative breast cancer. J Clin Oncol.

[CR6] Chia SK, Speers CH, D'yachkova Y, Kang A, Malfair-Taylor S, Barnett J, Coldman A, Gelmon KA, O'reilly SE, Olivotto IA (2007). The impact of new chemotherapeutic and hormone agents on survival in a population-based cohort of women with metastatic breast cancer. Cancer.

[CR7] Dafni U, Grimani I, Xyrafas A, Eleftheraki AG, Fountzilas G (2010). Fifteen-year trends in metastatic breast cancer survival in Greece. Breast Cancer Res Treat.

[CR8] Gennari A, Conte P, Rosso R, Orlandini C, Bruzzi P (2005). Survival of metastatic breast carcinoma patients over a 20-year period: a retrospective analysis based on individual patient data from six consecutive studies. Cancer.

[CR9] Giordano SH, Buzdar AU, Smith TL, Kau SW, Yang Y, Hortobagyi GN (2004). Is breast cancer survival improving?. Cancer.

[CR10] Holleczek B, Arndt V, Stegmaier C, Brenner H (2011). Trends in breast cancer survival in Germany from 1976 to 2008 - A period analysis by age and stage. Cancer Epidemiol.

[CR11] Lin NU, Carey LA, Liu MC, Younger J, Come SE, Ewend M, Harris GJ, Bullitt E, Van den Abbeele AD, Henson JW, Li X, Gelman R, Burstein HJ, Kasparian E, Kirsch DG, Crawford A, Hochberg F, Winer EP (2008). Phase II trial of lapatinib for brain metastases in patients with human epidermal growth factor 2-positive breast cancer. J Clin Oncol.

[CR12] Marty M, Cognetti F, Maraninchi D, Snyder R, Mauriac L, Tubiana-Hulin M, Chan S, Grimes D, Antón A, Lluch A, Kennedy J, O'Byrne K, Conte P, Green M, Ward C, Mayne K, Extra JM (2005). Randomized phase II trial of the efficacy and safety of trastuzumab combined with docetaxel in patients with human epidermal growth factor receptor 2-positive metastatic breast cancer administered as first line treatment: the M77001 study group. J Clin Oncol.

[CR13] Mauri D, Polyzos NP, Salanti G, Pavlidis N, Ioannidis JP (2008). Multiple-treatments meta-analysis of chemotherapy and targeted therapies in advanced breast cancer. J Natl Cancer Inst.

[CR14] Mehta RS, Barlow WE, Albain KS, Vandenberg TA, Dakhil SR, Tirumali NR, Lew DL, Hayes DF, Gralow JR, Livingston RB, Hortobagyi GN (2012). Combination anastrozole and fulvestrant in metastatic breast cancer. N Engl J Med.

[CR15] Mergenthaler U, Heymanns J, Köppler H, Thomalla J, van Roye C, Schenk J, Weide R (2011). Evaluation of psychosocial distress in patients treated in a community-based oncology group practice in Germany. Ann Oncol.

[CR16] Montemurro F, Rossi V, Geuna E, Valabrega G, Martinello R, Milani A, Aglietta M (2012). Current status and future perspectives in the endocrine treatment of postmenopausal, hormone receptor-positive metastatic breast cancer. Expert Opin Pharmacother.

[CR17] Mouridsen H, Gershanovich M, Sun Y, Perez-Carrion R, Boni C, Monnier A, Apffelstaedt J, Smith R, Sleeboom HP, Jaenicke F, Pluzanska A, Dank M, Becquart D, Bapsy PP, Salminen E, Snyder R, Chaudri-Ross H, Lang R, Wyld P, Bhatnagar A (2003). Phase III study of letrozole versus tamoxifen as first-line therapy of advanced breast cancer in postmenopausal women: analysis of survival and update of efficacy from the International Letrozole Breast Cancer Group. J Clin Oncol.

[CR18] Murphy CG, Morris PG (2012). Recent advances in novel targeted therapies for Her2-positive breast cancer. Anticancer Drugs.

[CR19] Nabholtz JM, Buzdar A, Pollak M, Harwin W, Burton G, Mangalik A, Steinberg M, Webster A, von Euler M (2000). Anastrozole is superior to tamoxifen as first-line therapy for advanced breast cancer in postmenopausal women: results of a North American multicenter randomized trial. Arimidex Study Group. J Clin Oncol.

[CR20] Office for National Statistics (ONS): **Cancer survival by cancer network, patients diagnosed in 1996–2009, followed up to 2010.** http://www.ons.gov.uk/ons/publications/re-reference-tables.html?edition=tcm%3A77-241176. Accessed 25 September 2013

[CR21] Onkopedia: **Leitlinien: Brustkrebs der Frau.** https://www.dgho-onkopedia.de/de/mein-onkopedia/leitlinien/brustkrebs-der-frau. Accessed 05 May 2014.

[CR22] Robertson JF, Lindemann JP, Llombart-Cussac A, Rolski J, Feltl D, Dewar J, Emerson L, Dean A, Ellis MJ (2012). Fulvestrant 500 mg versus anastrozole 1 mg for the first-line treatment of advanced breast cancer: follow-up analysis from the randomized FIRST study. Breast Cancer Res Treat.

[CR23] Rugo HS, Olopade O, DeMichele A, van 't Veer L, Buxton M, Hylton N, Yee D, Chien AJ, Wallace A, Lyandres J, Davis S, Sanil A, Berry D, Esserman L, I-SPY 2 Site PI's (2013). Veliparib/carboplatin plus standard neoadjuvant therapy for high-risk breast cancer: First efficacy results from the I-SPY 2 TRIAL.

[CR24] Schlesinger-Raab A, Eckel R, Engel J, Sauer H, Löhrs U, Molls M, Hölzel D (2005). Metastasiertes Mammakarzinom: Keine Lebensverlängerung seit 20 Jahren. Dtsch Arztebl.

[CR25] Sikov WM, Berry DA, Perou CM, Singh B, Cirrincione C, Tolaney S, Kuzma CS, Pluard TJ, Somlo G, Port E, Golshan M, Bellon JR, Collyar D, Hahn OM, Carey LA, Hudis C, Winer EP (2013). Impact of the addition of carboplatin (Cb) and/or bevacizumab (B) to neoadjuvant weekly paclitaxel (P) followed by dose-dense AC on pathologic complete response (pCR) rates in triple-negative breast cancer (TNBC): CALGB 40603 (Alliance).

[CR26] Slamon DJ, Clark GM, Wong SG, Levin WJ, Ullrich A, McGuire WL (1987). Human breast cancer: correlation of relapse and survival with amplification of the HER-2/neu oncogene. Science.

[CR27] Slamon DJ, Leyland-Jones B, Shak S, Fuchs H, Paton V, Bajamonde A, Fleming T, Eiermann W, Wolter J, Pegram M, Baselga J, Norton L (2001). Use of chemotherapy plus a monoclonal antibody against Her2 for metastatic breast cancer that overexpress Her2. N Engl J Med.

[CR28] Statistisches Bundesamt: **Todesursachen.** https://www.destatis.de/DE/ZahlenFakten/GesellschaftStaat/Gesundheit/Todesursachen/Tabellen/SterbefaelleInsgesamt.html. Accessed 09 May 2014

[CR29] Surveillance, Epidemiology and End Results Program (SEER 18, 2004–2010): **SEER Stat Fact Sheets. Breast Cancer.** http://seer.cancer.gov/statfacts/html/breast.html#survival. Accessed 09 December 2013.

[CR30] Swain SM, Kim SB, Cortés J, Ro J, Semiglazov V, Campone M, Ciruelos E, Ferrero JM, Schneeweiss A, Knott A, Clark E, Ross G, Benyunes MC, Baselga J (2013). Pertuzumab, trastuzumab and docetaxel for Her2-positive metastatic breast cancer (CLEOPATRA study): overall survival results from a randomised, double-blind, placebo-controlled, phase 3 study. Lancet Oncol.

[CR31] Tai P, Yu E, Vinh-Hung V, Cserni G, Vlastos G (2004). Survival of patients with metastatic breast cancer: twenty-year data from two SEER registries. BMC Cancer.

[CR32] Tevaarwerk AJ, Gray RJ, Schneider BP, Smith ML, Wagner LI, Fetting JH, Davidson N, Goldstein LJ, Miller KD, Sparano JA (2013). Survival in patients with metastatic recurrent breast cancer after adjuvant chemotherapy: little evidence of improvement over the past 30 years. Cancer.

[CR33] Tumorregister München: **Tumorstatistik: Überleben. C50.** Mammakarzinom (Frauen). http://www.tumorregister-muenchen.de/facts/surv/surv_C50f_G.pdf. Accessed 28 August 2013.

[CR34] Verma S, Miles D, Gianni L, Krop IE, Welslau M, Baselga J, Pegram M, Oh DY, Diéras V, Guardino E, Fang L, Lu MW, Olsen S, Blackwell K, EMILIA Study Group (2012). Trastuzumab emtansine for Her2-positive advanced breast cancer. N Engl J Med.

[CR35] von Minckwitz G, du Bois A, Schmidt M, Maass N, Cufer T, de Jongh FE, Maartense E, Zielinski C, Kaufmann M, Bauer W, Baumann KH, Clemens MR, Duerr R, Uleer C, Andersson M, Stein RC, Nekljudova V, Loibl S (2009). Trastuzumab beyond progression in human epidermal growth factor receptor 2-positive advanced breast cancer: a German breast group 26/breast international group 03–05 study. J Clin Oncol.

[CR36] Weide R, Mergenthaler U, Pandorf A, Arndt H, Heymanns J, Thomalla J, Köppler H (2009). Improved survival of patients with metastatic breast cancer in routine care: results of a retrospective study in a community-based oncology group practice 1995–2005. Onkologie.

[CR37] Weide R, Feiten S, Heymanns J, Thomalla J, van Roye C, Köppler H (2012). Die Behandlung von Patienten mit metastasierten soliden Tumoren in einer onkologischen Schwerpunktpraxis führt zu einem deutlich längeren Gesamtüberleben im Vergleich mit Registerdaten. Dtsch Med Wochenschr.

